# Differential Regulation of Dopamine Transporter Function and Location by Low Concentrations of Environmental Estrogens and 17β-Estradiol

**DOI:** 10.1289/ehp.0800026

**Published:** 2009-01-05

**Authors:** Rebecca A. Alyea, Cheryl S. Watson

**Affiliations:** Department of Biochemistry and Molecular Biology, University of Texas Medical Branch, Galveston, Texas, USA

**Keywords:** dopamine efflux, low concentrations, nongenomic, xenoestrogens

## Abstract

**Background:**

The effects of 17β-estradiol (E_2_) and xenoestrogens (XEs) on dopamine transport may have important implications for the increased incidence of neurologic disorders, especially in women during life stages characterized by frequent hormonal fluctuations.

**Objective:**

We examined low concentrations of XEs [dieldrin, endosulfan, *o*′*, p*′-dichlorodiphenyl-ethylene (DDE), nonylphenol (NP), and bisphenol A (BPA)] for nongenomic actions via action of membrane estrogen receptors (ERs).

**Methods:**

We measured activity of the dopamine transporter (DAT) by the efflux of ^3^H-dopamine in nontransfected nerve growth factor–differentiated PC12 rat pheochromocytoma cells expressing membrane DAT, ER-α, ER-β, and G-protein–coupled receptor 30. We used a plate immunoassay to monitor trafficking of these proteins.

**Results:**

All compounds at 1 nM either caused efflux or inhibited efflux, or both; each compound evoked a distinct oscillatory pattern. At optimal times for each effect, we examined different concentrations of XEs. All XEs were active at some concentration < 10 nM, and dose responses were all nonmonotonic. For example, 10^−14^ to 10^−11^ M DDE caused significant efflux inhibition, whereas NP and BPA enhanced or inhibited efflux at several concentrations. We also measured the effects of E_2_/XE combinations; DDE potentiated E_2_-mediated dopamine efflux, whereas BPA inhibited it. In E_2_-induced efflux, 15% more ER-α trafficked to the membrane, whereas ER-β waned; during BPA-induced efflux, 20% more DAT was trafficked to the plasma membrane.

**Conclusions:**

Low levels of environmental estrogen contaminants acting as endocrine disruptors via membrane ERs can alter dopamine efflux temporal patterning and the trafficking of DAT and membrane ERs, providing a cellular mechanism that could explain the disruption of physiologic neurotransmitter function.

Xenoestrogens (XEs) are environmental contaminants capable of mimicking the effects of endogenous estrogens, but usually not precisely. Thus, they can initiate more, different, and mistimed estrogen exposures that can lead to disruptions of estrogenic signaling (Watson et al. 2007b). XEs can enter the environment as manufacturing or agricultural by-products; these common human exposures are associated with a variety of reproductive and neurologic impairments (reviewed by Colborn 2004; Hotchkiss et al. 2008; McKinlay et al. 2008). One class of XEs includes a series of plastics monomers and detergents that are similar in structure. Bisphenol A (BPA), a monomer of polycarbonate plastics, is found in beverage bottles, canned food liners (50–100 nM), and epoxy dental sealants (Krishnan et al. 1993; Milligan et al. 1998; vom Saal and Hughes 2005). Nonylphenol (NP), also used in products of polymer manufacture, is additionally found in detergents, cleaning materials, and pesticides (Rudel et al. 2003). Many chlorinated pesticides [e.g., dieldrin, *o*′*, p*′-dichlorodiphenylethylene (DDE; an active metabolite of DDT), and endosulfan] can also behave as XEs. In addition, XEs break down slowly, so persistent deposits are found in the soil (Agency for Toxic Substances and Disease Registry 2002), thus exposing food supply plants and animals, which subsequently pass these exposures on to humans. Because XEs bioaccumulate in fat tissues, resulting in prolonged and escalating human exposures, the exposure levels causing deleterious health effects are actively debated.

Physiologic estrogens classically bind to the nuclear estrogen receptors ER-α and ER-β, eliciting transcription at genomic hormone response elements (Gruber et al. 2004). This process requires prolonged response times for synthesis and targeting of the macromolecules responsible for the hormonal changes. However, estrogens binding to the membrane form of these receptors (mERs) (reviewed by Watson and Gametchu 1999) are capable of eliciting rapid responses via nongenomic signaling and second-messenger systems at low (femtomolar to nanomolar) concentrations. Our laboratory previously demonstrated that XEs (Bulayeva and Watson 2004; Watson et al. 2005) and physiologic estrogens such as estradiol (E_2_), estrone (E_1_), and estriol (E_3_) (Watson et al. 2008) can act via mERs at very low concentration ranges in pituitary and breast cancer cells; these concentrations are far lower than those needed for classical nuclear responses. Thus, although physiologic estrogens such as E_2_, E_1_, E_3_, and environmental estrogen mimetics were long thought to be weak via the nuclear response pathway, we now know them to act quite potently via membrane-initiated response pathways (Watson et al. 2007a).

The dopamine transporter (DAT) is a selective transporter that primarily functions to stop dopamine signaling by removing dopamine (dopamine uptake) from the extracellular space after a stimulatory release of dopamine has occurred. Dopamine is subsequently brought into intracellular vesicles via vesicular monoamine transporters to protect it and maintain dopamine separation from other cellular molecules with which it might react. There it is stored for subsequent rapid signaling (reviewed by Torres et al. 2003). Estrogens and XEs may exert regulatory effects on multiple types of transporters (Toyohira et al. 2003). Our previous data have shown that E_2_ can affect both the DAT (Watson et al. 2006) and the serotonin transporter (Koldzic-Zivanovic et al. 2004) specifically, as shown by using selective inhibitors to measure the activity of these transporters.

Altered dopamine signaling is associated with specific pathological states such as Parkinson disease and schizophrenia, as well as drug addiction. Some studies have suggested a connection between XE exposures and altered dopamine signaling. High levels of endosulfan exposure (6 mg/kg for 22 days) resulted in decreased dopamine levels and impaired memory in Wistar rat pups (Lakshmana and Raju 1994). Polychlorinated biphenyls are known to inhibit vesicular trafficking of dopamine at micromolar concentrations and to reduce dopamine concentrations in rat synaptosomes, thereby resulting in increased extracellular dopamine (Bemis and Seegal 2004). Extremely high concentrations of BPA (above typical environmental contamination levels) cause rapid release of dopamine via the cyclic AMP/PKA pathway and N-type Ca^2+^ channels in PC12 pheochromocytoma cells (Yoneda et al. 2003). DDE, dieldrin, and endosulfan cause neuro toxicity (Bornman et al. 2007; Kitazawa et al. 2001; Naqvi and Vaishnavi 1993) in *in vivo* and *in vitro* systems. These effects of XEs might be due in part to their estrogenic effects via the nongenomic signaling pathways, although this has not been tested.

We recently used nerve growth factor (NGF)-differentiated PC12 cells, a well-characterized neuronal cell model, to demon strate that E_2_ inhibits dopamine uptake (Watson et al. 2006) and subsequently causes rapid dopamine efflux, primarily via the mER-α (Alyea et al. 2008). Here we report that very low concentrations of XEs also cause efflux of dopamine via the DAT, displaying nonconventional dose–response patterns and altered time phasing. We also show that combinations of the physiologic estrogen E_2_ with some XEs (which exposures of animals and humans often entail) can cause either potentiation or attenuation of E_2_-mediated dopamine efflux, depending on the estrogen mimetic coadministered. Finally, we report a functional link between the subcellular location of the ER subtypes and DAT resulting from BPA treatment compared with treatment with E_2_. These cellular events could explain the impact of XE exposures on dopamine efflux, including changes in neurotransmission, and some forms of functional endocrine disruption associated with neurotoxicity, especially in the context of diseases that are affected differentially between males and females.

## Materials and Methods

### Materials

We purchased fetal bovine serum (FBS) from Atlanta Biologicals (Lawrenceville, GA); equine serum from Gibco (Carlsbad, CA); ^3^H-dopamine from PerkinElmer (Waltham, MA); Scintiverse II from Fisher (Pittsburgh, PA); DDE and endosulfan from Ultra Scientific (North Kingstown, RI); and ABC (avidin:biotinylated enzyme complex) with alkaline phosphatase, *para*-nitrophenol phosphate, and levamisole from Vector Laboratories (Burlingame, CA). All other compounds, if not specified in text, were obtained from Sigma Chemical Co. (St. Louis, MO).

### PC12 cell culture

PC12 cells were grown in high-glucose, phenol red–free RPMI 1640 medium containing 5% FBS and 5% equine serum. For all experiments, cells were transferred to medium supplemented with 0.5% 4× charcoal-stripped FBS and 0.5% equine serum 48 hr before experimentation to minimize the effects of endogenous hormones. To promote differentiation and up-regulate DAT and mER-α, 20 ng/mL NGF-β was also added for 48 hr. Cells of passages 61–64 were used for these studies.

### Dopamine efflux assay

As described previously (Alyea et al. 2008), we adapted the method of Janowsky et al. (1998) to measure ^3^H-dopamine efflux using selective inhibitors to define the transporter. We plated PC12 cells on poly--lysine (10 μg/mL)-coated 48-well plates, deprived of endogenous hormones. We added uptake buffer (25 mM HEPES, 120 mM NaCl, 5 mM KCl, 2.5 mM CaCl_2_, 1.2 mM MgSO_4_, 1 μM pargyline, 2 mg/mL glucose) containing 0.2 mg/mL ascorbic acid and 50 nM desipramine (pH 7.4) with or without 1-(2-[bis(4-fluorophenyl)methoxy]ethyl)-4-(3-phenylpropyl) piperazine dihydro chloride (GBR 1290) for 60 min at 37°C. GBR 12909 (100 nM) was added for a 60-min preincubation to define selective efflux by DAT. ^3^H-Dopamine (20 nM) was loaded into the cells for 10 min before two washes in release buffer (25 mM HEPES, 120 mM NaCl, 5 mM KCl, 1.2 mM MgSO_4_, 1 μM pargyline, 2 mg/mL glucose, 0.2 mg/mL ascorbic acid, and 50 nM desipramine). We then added release buffer containing treatments with or without GBR 12909 and collected extracellular fluid at the indicated time points to assess efflux. Triplicate aliquots of each well were counted in 2 mL Scintiverse II scintillant using a Beckman LS600SE scintillation counter (Beckman Coulter Inc., Fullerton, CA) averaged to provide one sample (*n* = 1) per well. Specific efflux was defined by averaging the disintegrations per minute due to efflux in the presence of desipramine and GBR 12909, and then subtracting these values from the efflux observed with desipramine alone. We subtracted background (vehicle controls) matched for ethanol concentration and time from treatment groups and present the data as ^3^H-dopamine efflux per 10^6^ cells. Our rationale for using 1 nM E_2_ in combination with a range of concentrations for the XEs DDE and BPA was the prevalence of approximately 1 nM E_2_ as a physiologic concentration of total circulating E_2_ to which XEs would likely be added.

### Quantitative immunoplate assay

Briefly, PC12 cells were plated on poly--lysine (10 μg/mL)-coated 96-well plates at 5,000 cells/well, as previously described (Watson et al. 2006). Differentiated, serum-deprived cells were washed with phosphate-buffered saline (PBS) for 5 min, and treatments were added in the above uptake buffer with 50 nM dopamine for the indicated time points. Cells were fixed for 30 min at room temperature with 50 μL 2% paraformaldehyde and 0.2% gluteraldehyde with or without NP-40 (nonyl-phenol ethoxylate) for permeabilized and non-permeabilized cells, respectively. Cells were then washed twice (5 min each) with PBS and blocked with 0.1% fish gelatin/PBS for 45 min at 22°C. We added diluted primary antibodies to ER-α (1:1,000; Mc-20 and sc-542; Santa Cruz Biotechnology, Inc., Santa Cruz, CA), ER-β (1:1,500; clone 9.88, E1276; Sigma), G-protein–coupled receptor 30 (GPR30; 1:1,000; NLS4271; Novus, Littleton, CO), or DAT (1:2,000; W-17, sc-33056; Santa Cruz Biotechnology) overnight at 4°C; substitution of 2 μg anti-clathrin antibody provided a control for cell permeabilization (Campbell and Watson 2001). Cells were washed three times in PBS, incubated in appropriate biotinylated secondary antibodies for 1 hr, and then washed three times before a 1-hr incubation with ABC-alkaline phosphatase solution. After cells were washed five times with PBS, the substrate *para*-nitrophenol phosphate plus 0.5 mM levamisole was added in 100 mM sodium bicarbonate solution for 50 min at 37°C. Plates were read at 405 nm absorbance (A_405_), rinsed and stained with 0.1% crystal violet for 30 min at room temperature, washed with double-distilled H_2_O, and dried overnight. We read dye (extracted from each well with 50 μL 10% acetic acid) at A_590_ and used this to estimate cell number per well. Data are plotted as percentage of control levels (A_405_/A_590_).

### Statistics

We repeated each experimental set three times on different cell preparations, and we report the number for each treatment in the figure legends. We used analysis of variance to indicate whether further testing of individual data points for significance from their control was warranted. We performed statistical analyses for all assays using SigmaStat software (Systat Software, Inc., Chicago, IL) and set statistical significance at *p* < 0.05.

## Results

**Figure 1:**
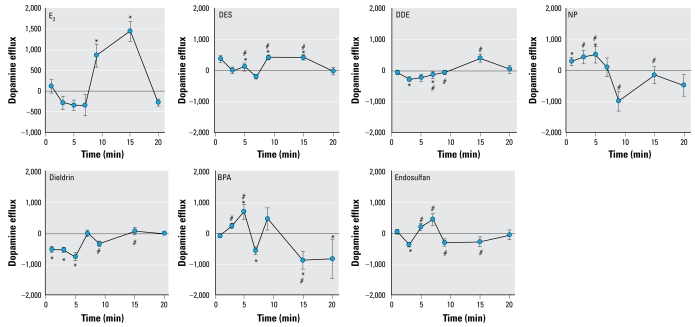
Effects of 10^−9^ M E_2_ (*n* = 18), DES (*n* = 23), DDE (*n* = 14), NP (*n* = 18), dieldrin (*n* = 14), BPA (*n* = 14), and endosulfan (*n* = 18) on the dopamine efflux time course. The 10^−9^ M E_2_ time course is reproduced from Alyea et al. (2008), with permission of *Journal of Neurochemistry*, and is included here for purposes of comparison. Values are means and SEs. Points above the zero point line indicate a positive efflux of dopamine from the cells. *p < 0.05 compared with control. ^#^*p* < 0.05 compared with E_2_ treatment.

We have previously demonstrated that 10^−9^ M E_2_ causes rapid dopamine efflux of preloaded ^3^H-dopamine from PC12 cells (Alyea et al. 2008). Figure 1 shows time courses for E_2_ compared with each XE that we studied at this concentration. Both E_2_ and diethyl stilbestrol (DES) displayed peak dopamine efflux at 9–15 min, followed by a return to baseline. NP and BPA caused rapid dopamine efflux within 5 min, followed by a return to baseline for NP and inhibited dopamine efflux for BPA. The pesticides DDE, dieldrin, and endosulfan did not cause dopamine efflux, but dopamine efflux inhibition occurred as early as 1 min for dieldrin and 3 min for DDE and endo-sulfan, followed by continuous baseline activity. Interestingly, our results suggest that the compounds that belong to the same functional use/chemical structure class [grouped as the pesticides (DDE, endosulfan, dieldrin), plastics compounds (BPA, NP), and E_2_ along with the pharmaceutical estrogen DES (considered to be largely equivalent in actions by genomic tests)] cause similar temporal and stimulatory/inhibitory regulation of dopamine efflux. The rapidity with which all tested compounds in these studies either cause or inhibit dopamine efflux implies that nongenomic actions are involved.

**Figure 2:**
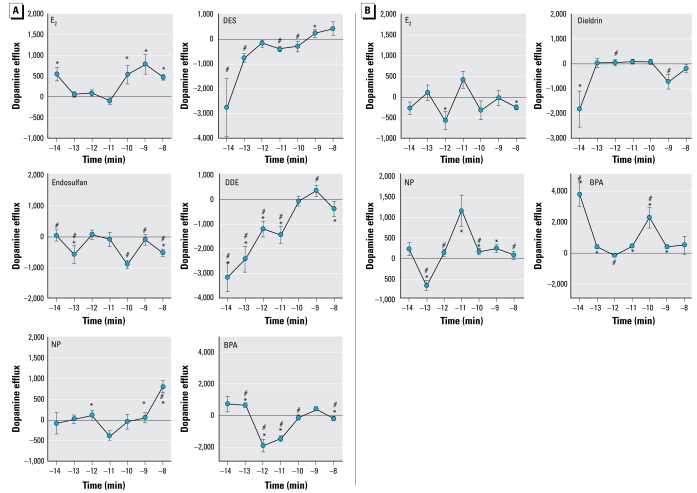
Concentration-dependent dopamine efflux patterns for E_2_ and XEs at 9 (*A*) and 5 min (*B*), using optimal time points for each compound chosen from the 10^−9^ M time course (Figure 1). (*A*) A 9-min dopamine efflux for E_2_, DES, endosulfan, DDE, NP, and BPA at concentrations ranging from 10^−14^ to 10^−9^ M. (*B*) A 5-min dopamine efflux for E_2_, dieldrin, NP, and BPA at concentrations ranging from 10^−14^ to 10^−9^ M. Values are means and SEs; numbers per treatment are as follows: E_2_, *n* = 18; dieldrin, *n*= 12; DES, *n*= 18; endosulfan, *n*= 12; DDE, *n*= 12; BPA, *n*= 23; NP, *n*= 15. Points above the zero point line indicate a positive efflux of dopamine from the cells. *p < 0.05 compared with control. #*p* < 0.05 compared with E_2_ treatment.

Extensive dose–response curves for each compound at optimal time points, including exposure-relevant or physiologic/treatment-relevant doses in the case of endogenous/pharmaceutical estrogens, are important to assess both for practical applicability of the results and to fully characterize the nongenomic actions of XEs, which we have shown in the past to elicit nonconventional nonmonotonic dose responses at low levels (Bulayeva et al. 2002; Bulayeva and Watson 2004; Wozniak et al. 2005). At 9 min, E_2_ caused dopamine efflux at the lowest concentration tested (10^−14^M) and at the higher but still physiologic concentrations (10^−10^ to 10^−8^ M), but not at the intermediate concentrations (Figure 2A). Although all compounds exhibited multiple dose-induced stages of dopamine efflux, DES, endosulfan, and BPA caused dopamine efflux inhibition at quite low concentrations at 9 min. NP and DES also caused efflux at some of the higher concentrations tested. We also tested E_2_, dieldrin, NP, and BPA dopamine efflux dose responses at 5 min (Figure 2B) to examine the different flux states caused by these compounds at optimally active response times (for enhancement or inhibition) for each. E_2_ inhibited dopamine efflux at 10^−12^ and 10^−8^ M. NP inhibited dopamine efflux at 10^−13^ M, followed by a large peak of dopamine efflux at 10^−11^ M and continuing modest efflux through 10^−10^ to 10^−9^ M concentrations. Dieldrin inhibited dopamine efflux at both 10^−14^ and 10^−9^ M concentrations, whereas all other concentrations did not modify activity from basal. Interestingly, 10^−14^ M BPA at this time point caused a 3-fold higher dopamine efflux than at any concentration or time point tested for E_2_.

**Figure 3:**
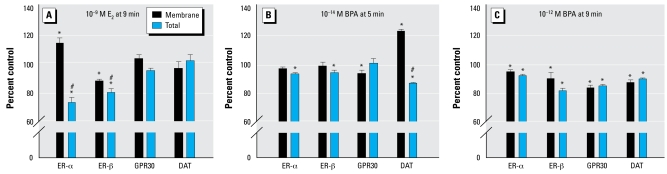
Quantitative plate assay measuring immunoreactive protein levels for plasma membrane and total ER-α, ER-β, GPR30, and DAT after treatment with E_2_ for 9 min (*A*), or BPA for 5 (*B*) or 9 min (*C*). (*A*) 10^−9^ M E_2_ for 9 min (dopamine efflux was significantly increased at this time point and concentration). (*B*) 10^−14^ M BPA for 5 min (DA efflux was significantly increased at this time point and concentration). (*C*) 10^−12^ M BPA for 9 min (DA efflux was significantly inhibited at this time point and concentration). (*A*) is modified from Alyea et al. (2008) with permission of *Journal of Neurochemistry.* Values are means and SEs; *n* = 24 for all compounds. Points above the zero point line indicate a positive efflux of dopamine from the cells. *p < 0.05 compared with control. ^#^*p* < 0.05 compared with membrane, shown here for comparison with changes caused by BPA alone.

Our previous results (Alyea et al. 2008) showed that ER-α is the predominant mediator of E_2_-mediated dopamine efflux, with some possible inhibitory contributions from ER-β and GPR30. Therefore, we next investigated the effects of E_2_ and BPA on the subcellular location of all known ER subtypes and the DAT itself in treated cells. We compared this with untreated levels (100%) at times and concentrations representing their maximum effects on efflux enhancement or inhibition. We assessed the membrane versus total cellular levels of these proteins in our quantitative immunoreactive plate assay (Figure 3), which uses the permeabilized status of the cells to distinguish these values (Campbell and Watson 2001). At 9 min, a 10^−9^ M E_2_ treatment increased membrane levels of ER-α (a stimulator of efflux at this time and concentration) and decreased membrane ER-β (an inhibitor of this response), whereas total levels of these proteins declined in the cells (Figure 3A); the levels of GPR30 and DAT remained unchanged in both compartments. However, 10^−14^ M BPA at 5 min (Figure 3B), which correlates with the highest level of dopamine efflux, did not change levels of membrane ER-α or ER-β (with very slight overall declines of these proteins) and slightly decreased membrane GPR30. Interestingly, the most robust change in this scenario was an increased membrane level of DAT, which could itself explain increased dopamine-pumping capacity. At 9 min, 10^−12^ M BPA (Figure 3C), a time point and concentration that inhibited dopamine efflux, caused trafficking of all three ERs and the DAT away from the plasma membrane, the combination of which would be expected to diminish the ability of the cell to pump dopamine out. These results suggest that changes in BPA-mediated dopamine efflux are due to distinctly different mechanisms than E_2_-mediated changes in dopamine efflux.

**Figure 4:**
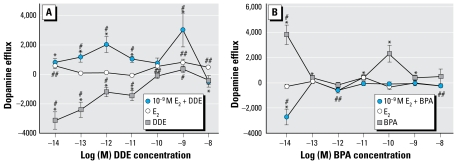
Effects of physiologic levels of E_2_ (10^−9^ M) combined with multiple concentrations of DDE or BPA measured by dopamine efflux assay. (*A*) E_2_ plus increasing concentrations of DDE during a 9-min dopamine efflux assay compared with E_2_-mediated dopamine efflux and DDE-mediated dopamine efflux. (*B*) E_2_ plus increasing concentrations of BPA during a 5-min dopamine efflux assay compared with E_2_-mediated dopamine efflux and BPA-mediated dopamine efflux. Values are means and SEs; numbers per treatment are as follows: E_2_, *n* = 18; BPA, *n* = 14; DDE, *n* = 14; DDE + E_2_, *n* = 15; BPA + E_2_, *n* = 15. Points above the zero point line indicate a positive efflux of dopamine from the cells. *p < 0.05 compared with control for combinations. ^#^*p* < 0.05 compared with E_2_ treatment. ^##^*p* < 0.05 compared with control for E_2_.

In our natural environment, exposure to these compounds is combinatorial, due to not only the presence of multiple compounds simultaneously but also the presence of endogenous steroids. Therefore, we examined combinations for a limited number of these cases by comparing single exposures with a physiologic dose of E_2_ versus combinations of E_2_ with DDE (Figure 4A) and BPA (Figure 4B) at multiple concentrations. Alone, DDE at 9 min dramatically inhibited dopamine efflux at all concentrations ≤ 10^−11^ M, whereas E_2_ caused dopamine efflux at 10^−14^ M and at ≥ 10^−10^ M (as shown previously in Figure 2A with different optimized scales for the response). In combination with 10^−9^ M E_2_, DDE caused dopamine efflux at 10^−14^ to 10^−11^ M and at 10^−9^ M, clearly different (enhancing the response) compared with either compound alone (Figure 4A). The combination magnitude of the response was a 3-fold increase above DDE alone. The dose–response curve was thus shifted from inhibitory to a more than additive heightening of dopamine efflux. Interestingly, this was not the case with BPA in combination with E_2_ (Figure 4B), where we saw inhibition of dopamine efflux at all stimulatory concentrations for either compound alone. At 5 min, 10^−14^ M BPA caused the greatest level of dopamine efflux, which was substantially reversed by combination with 10^−9^ M E_2_. Only 10^−13^ M BPA still caused efflux (slightly) in the presence of physiologic levels of E_2_, which was not significantly different from BPA or E_2_ alone at that concentration. Therefore, these two different XEs in combination with E_2_ altered dopamine efflux states compared with their singular effects in dramatically different ways.

## Discussion

Epigenetic events caused by unknown neuro-toxins have been implicated in initiating malfunction and degeneration of dopaminergic neurons, and many have speculated that environmental contaminants such as XEs are linked to the increasing incidence of neurologic diseases and disorders (Colborn 2004; Prasad et al. 1999). This is the first study to examine the nongenomic estrogenic effects of some of these xeno biotics on dopamine efflux at low, environmentally relevant exposure levels, comparing details of the functions at the cellular level with the presence and location of the cellular proteins involved (DAT and the different ERs).

On the basis of the dopamine efflux responses to these different estrogens (time courses and concentrations curves), we observed some similar patterns within subclasses of these chemicals. First, the group consisting of E_2_ and DES (a nonsteroidal synthetic pharmaceutical) caused rapid dopamine efflux, similarly peaking at 9–15 min. DES also has genomic actions similar to those of E_2_ (Arnold et al. 1996). However, the DES dose–response pattern at 9 min did not resemble that for E_2_ at 9 min, so our studies show some differences between these compounds. Many studies in the past have used DES as a positive control for estrogenic responses because of the similar functional responses in many past animal and human studies (Fail et al. 1998; Fang et al. 2001; Kwon et al. 2007; vom Saal et al. 1997; Yang et al. 2008). However, DES has a higher relative binding affinity for recombinant human ER-α and ER-β than does E_2_ (Nikov et al. 2000). In a previous study (Alyea et al. 2008), we showed that although ER-α is the predominant mediator of E_2_-mediated dopamine efflux, ER-β and GPR30 could have inhibitory roles. Therefore, differential DES (or other XE) binding to ER-β and GPR30 in our cell model could affect estrogen-mediated dopamine efflux.

NP and BPA both caused rapid dopamine efflux within 5 min, followed by basal and then inhibitory responses. However, these XE-induced dopamine efflux patterns at 3–5 min differed significantly from the effects of E_2_ (which were inhibitory). This (and other examples from this series of experiments) demonstrates that XEs, causing efflux in distinctly different temporal patterns compared with E_2_, could cause altered patterns of response in combination. We have previously reported that some XEs also elicit responses differentially in pituitary cells (Bulayeva and Watson 2004; Wozniak et al. 2005), activating multiple temporally regulated kinase pathways that undoubtedly interact with one another, causing potential points of opposing or mis-regulation. An alternate receptor-binding profile for XEs could also modify the inclusion or extent of use of these multiple XE-induced signaling pathways. Such actions on a background of responses to physiologic levels of E_2_ or other estrogens exemplify how these compounds could disrupt the endocrine functions of humans and animals.

The third “use and structure class” of compounds that we studied were polychlorinated pesticides or their metabolites (DDE, endosulfan, and dieldrin). They caused rapid inhibition of dopamine efflux at the lower concentrations. Some organochlorine pesticides, such as DDE and dieldrin, have been shown to cause increased dopamine, DAT, and vesicular monoamine transporter levels *in vivo* (Richardson et al. 2006; Sava et al. 2007). *In utero* exposure to dieldrin leads to a delayed and enhanced vulnerability of dopamine neurons to the parkinsonism-inducing neurotoxin MPTP (1-methyl-4-phenyl-1,2,5,6-tetrahydropyridine), which is oxidized to MPP^+^ (1-methyl-4-phenyl-pyridinium), the primary constituent of the herbicide cyperquat (Richardson et al. 2006). These effects are greater in males than in females (Richardson et al. 2006), suggesting a protective effect of physiologic estrogens.

Our extensive range of dose–response curves, at two time points for some compounds, revealed that E_2_ and all XEs that we studied can cause dopamine efflux changes at low, environmentally relevant concentrations. We also show that at some of these rapid time points and low concentrations, these compounds caused significant inhibition of dopamine efflux. Only a careful mapping of the response effects of combinations of these and other estrogenic compounds (physiologic, pharmaceutical, industrial, agricultural, and dietary estrogens) will fully elucidate their aggregate potential for influencing dopamine efflux and thus contributing to adverse neurologic outcomes.

Although DDE alone did not cause dopamine efflux, it caused potentiation of dopamine efflux in the presence of physiologic levels of E_2_. Recently, Hatcher et al. (2008) reported that higher concentrations of DDE *in vitro* (although with documented lower levels actually reaching the brain tissue) matched human postmortem levels in Parkinson disease patients; these levels inhibit dopamine uptake and cause synaptosomal and vesicular release of dopamine without increasing intracellular oxidative stress. Increasing intra-cellular dopamine concentrations (by depleting vesicular stores) can promote dopamine efflux by exchange diffusion (Fischer and Cho 1979). It is possible that by combining DDE and E_2_ we are increasing intra cellular dopamine to the point that the mechanism shifts from efflux inhibition (or uptake) to efflux. This is only one of many possible mechanisms that should be explored for combinations of XEs and physiologic estrogens, or other XEs.

By examining the subcellular location of DAT and several ERs, we addressed how protein trafficking of important mediators might explain the functional effects of estrogens, including XEs, on dopamine transport. A mechanistic link can be made between the dopamine efflux functional state and the cellular location of the ERs. Membrane ER-α either is increased or stays in the membrane when efflux occurs. However, at a concentration and time point when BPA inhibits dopamine efflux, ER-α leaves the membrane. BPA-mediated dopamine efflux results in trafficking of the inhibitory GPR30 from the plasma membrane, but under conditions where BPA inhibits dopamine efflux, all three ERs (both stimulatory and inhibitory of this response) leave the plasma membrane. The increased levels of membrane DAT caused by efflux-producing BPA treatments suggest that BPA-mediated dopamine efflux involves distinctly different intermediary mechanisms compared with E_2_. Compounds such as BPA that increase DAT numbers at the membrane have been reported to be pathological because increased DATs in the membrane leads to increased toxin susceptibility (McArthur et al. 2007). Although both E_2_ and BPA cause dopamine efflux, E_2_ (which does not act by increasing DAT in the membrane) is reported to be neuroprotective (McEwen and Alves 1999). Therefore, BPA could cause increased vulnerability to dopamine-related diseases by increasing the level of functional DAT at the plasma membrane.

Women are abusing stimulants, such as amphetamine, methamphetamine, and cocaine, at an increasing rate. Although drug addiction and abuse rates involve many social factors, females display an association between sex steroid levels and drug behavioral and neurochemical effects (Evans 2007). Amphetamines and methamphetamines also cause dopamine efflux via the DAT (Volz et al. 2007). DAT displays sex differences; females have more DAT in the striatum than men (Mozley et al. 2001) although men experience higher amphetamine-stimulated striatal dopa mine release (Munro et al. 2006), perhaps a function of having a lower baseline unaffected by estrogens. Human females have been reported to experience increased euphoric feeling from these drugs during the follicular phase of the menstrual cycle, which is correlated with the highest E_2_ levels (White et al. 2002). If XEs are capable of further increasing dopamine efflux by increasing functioning DAT in the membranes, then they could be further exacerbating the effects of both physiologic estrogens and drugs of abuse, perhaps with behavioral consequences. In rodent models, prenatal and neonatal exposure to BPA leads to enhanced sensitivity to rewarding effects of methamphetamine (Suzuki et al. 2003) and morphine (Miyatake et al. 2006). It remains to be seen whether human XE exposure during specific developmental stages is associated with an increased vulnerability to drug addictions later in life, which might be especially relevant to women. If XEs can act this potently via nongenomic signaling pathways, then the current allowable and very prevalent contamination levels in our water and food supplies may lead to significant endocrine disruption and a variety of human diseases, including those affecting behavior.

## References

[b1-ehp-117-778] Agency for Toxic Substances and Disease Registry (2002). Toxicological Profile for Aldrin and Dieldrin.

[b2-ehp-117-778] Alyea RA, Laurence SE, Kim SH, Katzenellenbogen BS, Katzenellenbogen JA, Watson CS (2008). The roles of membrane estrogen receptor subtypes in modulating dopamine transporters in PC-12 cells. J Neurochem.

[b3-ehp-117-778] Arnold SF, Klotz DM, Collins BM, Vonier PM, Guillette LJ, McLachlan JA (1996). Synergistic activation of estrogen receptor with combinations of environmental chemicals. Science.

[b4-ehp-117-778] Bemis JC, Seegal RF (2004). PCB-induced inhibition of the vesicular monoamine transporter predicts reductions in synaptosomal dopamine content. Toxicol Sci.

[b5-ehp-117-778] Bornman MS, Pretorius E, Marx J, Smit E, van der Merwe CF (2007). Ultrastructural effects of DDT, DDD, and DDE on neural cells of the chicken embryo model. Environ Toxicol.

[b6-ehp-117-778] Bulayeva NN, Watson CS (2004). Xenoestrogen-induced ERK 1 and 2 activation via multiple membrane-initiated signaling pathways. Environ Health Perspect.

[b7-ehp-117-778] Campbell CH, Watson CS (2001). A comparison of membrane vs. intracellular estrogen receptor-alpha in GH(3)/B6 pituitary tumor cells using a quantitative plate immunoassay. Steroids.

[b8-ehp-117-778] Colborn T (2004). Neurodevelopment and endocrine disruption. Environ Health Perspect.

[b9-ehp-117-778] Evans SM (2007). The role of estradiol and progesterone in modulating the subjective effects of stimulants in humans. Exp Clin Psychopharmacol.

[b10-ehp-117-778] Fail PA, Chapin RE, Price CJ, Heindel JJ (1998). General, reproductive, developmental, and endocrine toxicity of boronated compounds. Reprod Toxicol.

[b11-ehp-117-778] Fang H, Tong W, Shi LM, Blair R, Perkins R, Branham W (2001). Structure-activity relationships for a large diverse set of natural, synthetic, and environmental estrogens. Chem Res Toxicol.

[b12-ehp-117-778] Fischer JF, Cho AK (1979). Chemical release of dopamine from striatal homogenates: evidence for an exchange diffusion model. J Pharmacol Exp Ther.

[b13-ehp-117-778] Gruber CJ, Gruber DM, Gruber IM, Wieser F, Huber JC (2004). Anatomy of the estrogen response element. Trends Endocrinol Metab.

[b14-ehp-117-778] Hatcher JM, Delea KC, Richardson JR, Pennell KD, Miller GW (2008). Disruption of dopamine transport by DDT and its metabolites. Neurotoxicology.

[b15-ehp-117-778] Hotchkiss AK, Rider CV, Blystone CR, Wilson VS, Hartig PC, Ankley GT (2008). Fifteen years after “Wingspread”—environmental endocrine disrupters and human and wildlife health: where we are today and where we need to go. Toxicol Sci.

[b16-ehp-117-778] Janowsky A, Neve K, Eshleman AJ (1998). Uptake and release of neurotransmitters. Curr Protocols Neurosci.

[b17-ehp-117-778] Kitazawa M, Anantharam V, Kanthasamy AG (2001). Dieldrin-induced oxidative stress and neurochemical changes contribute to apoptopic cell death in dopaminergic cells. Free Radic Biol Med.

[b18-ehp-117-778] Koldzic-Zivanovic N, Seitz PK, Watson CS, Cunningham KA, Thomas ML (2004). Intracellular signaling involved in estrogen regulation of serotonin reuptake. Mol Cell Endocrinol.

[b19-ehp-117-778] Krishnan AV, Stathis P, Permuth SF, Tokes L, Feldman D (1993). Bisphenol-A: an estrogenic substance is released from polycarbonate flasks during autoclaving. Endocrinology.

[b20-ehp-117-778] Kwon JH, Katz LE, Liljestrand HM (2007). Modeling binding equilibrium in a competitive estrogen receptor binding assay. Chemosphere.

[b21-ehp-117-778] Lakshmana MK, Raju TR (1994). Endosulfan induces small but significant changes in the levels of noradrenaline, dopamine and serotonin in the developing rat brain and deficits in the operant learning performance. Toxicology.

[b22-ehp-117-778] McArthur S, Murray HE, Dhankot A, Dexter DT, Gillies GE (2007). Striatal susceptibility to a dopaminergic neurotoxin is independent of sex hormone effects on cell survival and DAT expression but is exacerbated by central aromatase inhibition. J Neurochem.

[b23-ehp-117-778] McEwen BS, Alves SE (1999). Estrogen actions in the central nervous system 1. Endocr Rev.

[b24-ehp-117-778] McKinlay R, Plant JA, Bell JN, Voulvoulis N (2008). Calculating human exposure to endocrine disrupting pesticides via agricultural and non-agricultural exposure routes. Sci Total Environ.

[b25-ehp-117-778] Milligan SR, Balasubramanian AV, Kalita JC (1998). Relative potency of xenobiotic estrogens in an acute *in vivo* mammalian assay. Environ Health Perspect.

[b26-ehp-117-778] Miyatake M, Miyagawa K, Mizuo K, Narita M, Suzuki T (2006). Dynamic changes in dopaminergic neurotransmission induced by a low concentration of bisphenol-A in neurones and astrocytes. J Neuroendocrinol.

[b27-ehp-117-778] Mozley LH, Gur RC, Mozley PD, Gur RE (2001). Striatal dopamine transporters and cognitive functioning in healthy men and women. Am J Psychiatry.

[b28-ehp-117-778] Munro CA, McCaul ME, Wong DF, Oswald LM, Zhou Y, Brasic J (2006). Sex differences in striatal dopamine release in healthy adults. Biol Psychiatry.

[b29-ehp-117-778] Naqvi SM, Vaishnavi C (1993). Bioaccumulative potential and toxicity of endosulfan insecticide to non-target animals. Comp Biochem Physiol C.

[b30-ehp-117-778] Nikov GN, Hopkins NE, Boue S, Alworth WL (2000). Interactions of dietary estrogens with human estrogen receptors and the effect on estrogen receptor-estrogen response element complex formation. Environ Health Perspect.

[b31-ehp-117-778] Prasad KN, Cole WC, Kumar B (1999). Multiple antioxidants in the prevention and treatment of Parkinson’s disease. J Am Coll Nutr.

[b32-ehp-117-778] Richardson JR, Caudle WM, Wang M, Dean ED, Pennell KD, Miller GW (2006). Developmental exposure to the pesticide dieldrin alters the dopamine system and increases neurotoxicity in an animal model of Parkinson’s disease. FASEB J.

[b33-ehp-117-778] Rudel RA, Camann DE, Spengler JD, Korn LR, Brody JG (2003). Phthalates, alkylphenols, pesticides, polybrominated diphenyl ethers, and other endocrine-disrupting compounds in indoor air and dust. Environ Sci Technol.

[b34-ehp-117-778] Sava V, Velasquez A, Song S, Sanchez-Ramos J (2007). Dieldrin elicits a widespread DNA repair and antioxidative response in mouse brain. J Biochem Mol Toxicol.

[b35-ehp-117-778] Suzuki T, Mizuo K, Nakazawa H, Funae Y, Fushiki S, Fukushima S (2003). Prenatal and neonatal exposure to bisphenol-A enhances the central dopamine d1 receptor-mediated action in mice: enhancement of the methamphetamine-induced abuse state. Neuroscience.

[b36-ehp-117-778] Torres GE, Gainetdinov RR, Caron MG (2003). Plasma membrane monoamine transporters: structure, regulation and function. Nat Rev Neurosci.

[b37-ehp-117-778] Toyohira Y, Utsunomiya K, Ueno S, Minami K, Uezono Y, Yoshimura R (2003). Inhibition of the norepinephrine transporter function in cultured bovine adrenal medullary cells by bisphenol A. Biochem Pharmacol.

[b38-ehp-117-778] Volz TJ, Fleckenstein AE, Hanson GR (2007). Methamphetamine-induced alterations in monoamine transport: implications for neurotoxicity, neuroprotection and treatment. Addiction.

[b39-ehp-117-778] vom Saal FS, Hughes C (2005). An extensive new literature concerning low-dose effects of bisphenol A shows the need for a new risk assessment. Environ Health Perspect.

[b40-ehp-117-778] vom Saal FS, Timms BG, Montano MM, Palanza P, Thayer KA, Nagel SC (1997). Prostate enlargement in mice due to fetal exposure to low doses of estradiol or diethylstilbestrol and opposite effects at high doses. Proc Natl Acad Sci USA.

[b41-ehp-117-778] Watson CS, Alyea RA, Hawkins BE, Thomas ML, Cunningham KA, Jakubas AA (2006). Estradiol effects on the dopamine transporter—protein levels, subcellular location, and function. J Mol Signal.

[b42-ehp-117-778] Watson CS, Alyea RA, Jeng YJ, Kochukov MY (2007a). Nongenomic actions of low concentration estrogens and xenoestrogens on multiple tissues. Mol Cell Endocrinol.

[b43-ehp-117-778] Watson CS, Bulayeva NN, Wozniak AL, Alyea RA (2007b). Xenoestrogens are potent activators of nongenomic estrogenic responses. Steroids.

[b44-ehp-117-778] Watson CS, Bulayeva NN, Wozniak AL, Finnerty CC (2005). Signaling from the membrane via membrane estrogen receptor-α: estrogens, xenoestrogens, and phyto-estrogens. Steroids.

[b45-ehp-117-778] Watson CS, Gametchu B (1999). Membrane-initiated steroid actions and the proteins that mediate them. Proc Soc Exp Biol Med.

[b46-ehp-117-778] Watson CS, Jeng YJ, Kochukov MY (2008). Nongenomic actions of estradiol compared with estrone and estriol in pituitary tumor cell signaling and proliferation. FASEB J.

[b47-ehp-117-778] White TL, Justice AJ, de Wit H (2002). Differential subjective effects of d-amphetamine by gender, hormone levels and menstrual cycle phase. Pharmacol Biochem Behav.

[b48-ehp-117-778] Wozniak AL, Bulayeva NN, Watson CS (2005). Xenoestrogens at picomolar to nanomolar concentrations trigger membrane estrogen receptor-alpha-mediated Ca^2+^ fluxes and prolactin release in GH3/B6 pituitary tumor cells. Environ Health Perspect.

[b49-ehp-117-778] Yang L, Lin L, Weng S, Feng Z, Luan T (2008). Sexually disrupting effects of nonylphenol and diethylstilbestrol on male silver carp (Carassius auratus) in aquatic microcosms. Ecotoxicol Environ Saf.

[b50-ehp-117-778] Yoneda T, Hiroi T, Osada M, Asada A, Funae Y (2003). Non-genomic modulation of dopamine release by bisphenol-A in PC12 cells. J Neurochem.

