# Errata

**Published:** 2009-10

In the article by Alyea and Watson [Environ Health Perspect 117:778–783 (2009)], the *x*-axis labels in Figure 2 were incorrect. The corrected figure appears below.

*EHP* apologizes for the error.

## Figures and Tables

**Figure 2:**
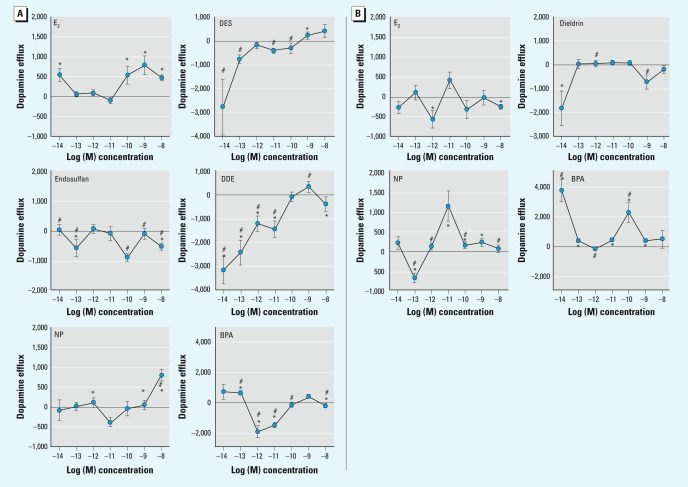
Concentration-dependent dopamine efflux patterns for E_2_ and XEs at 9 (*A*) and 5 min (*B*), using optimal time points for each compound chosen from the 10^–9^ M time course (Figure 1). (*A*) A 9-min dopamine efflux for E_2_, DES, endosulfan, DDE, NP, and BPA at concentrations ranging from 10^–14^ to 10^–9^ M. (*B*) A 5-min dopamine efflux for E_2_, dieldrin, NP, and BPA at concentrations ranging from 10^–14^ to 10^−9^ M. Values are means and SEs; numbers per treatment are as follows: E_2_, *n* = 18; dieldrin, *n* = 12; DES, *n* = 18; endosulfan, *n* = 12; DDE, *n* = 12; BPA, *n* = 23; NP, *n* = 15. Points above the zero point line indicate a positive efflux of dopamine from the cells. *p < 0.05 compared with control. #*p* < 0.05 compared with E_2_ treatment.

